# Authentic Leadership in Lithuanian Healthcare: A Qualitative Exploratory Study of Health Managers’ Views and Experiences

**DOI:** 10.15388/Amed.2026.33.1.6

**Published:** 2026-07-22

**Authors:** Miglė Lazinkaitė, Mark Avery, Richard Olley, Aurima Stankūnienė, Katarzyna Czabanowska, Mindaugas Stankūnas

**Affiliations:** 1 Vilnius City Public Health Bureau, Vilnius, Lithuania E-mail: ORCID ID; 2 School of Applied Psychology, Griffith University, Brisbane, Australia E-mail: ORCID ID; 3 School of Medicine and Dentistry, Griffith University, Brisbane, Australia E-mail: ORCID ID; 4 Department of Drug Technology and Social Pharmacy, Lithuanian University of Health Sciences, Kaunas, Lithuania E-mail: ORCID ID; 5 Department of International Health, Care and Public Health Research Institute (CAPHRI), Maastricht University, Maastricht, The Netherlands E-mail: ORCID ID; 6 Lithuanian University of Health Sciences, Kaunas, Lithuania School of Applied Psychology, Griffith University, Brisbane, Australia E-mail: ORCID ID

**Keywords:** authentic leadership, healthcare, management, Lithuania, autentiškas vadovavimas, sveikatos priežiūra, vadyba, Lietuva

## Abstract

Authentic Leadership (AL) is a value-based approach to leadership that focuses on self-awareness, relational transparency, balanced processing, and an internalized moral perspective. AL nurtures a supportive, high-trust work environment that boosts team cohesion, productivity, and ethical standards. In the healthcare workforce, the challenges and demands of person-centred care are significant, making AL a valuable opportunity to enhance the effectiveness of leadership and management. This study explored the key constructs and opportunities of AL within the context of the Lithuanian health system.

A qualitative interpretative phenomenological approach was used to conduct semi-structured interviews with senior healthcare managers in Lithuania. The goal was to explore how these leaders understood and practiced AL. Thematic analysis was performed on the transcriptions of study participants’ semi-structured interviews about perceptions of AL’s four theoretical dimensions.

The findings identified that Lithuanian healthcare leaders view self-awareness, moral integrity, and adaptability as central to effective leadership. Leaders emphasized self-reflection, openness to feedback, and moral consistency as crucial to their roles, enabling them to manage complex ethical responsibilities and strengthen organizational trust. AL has been shown to promote resilience in dynamic healthcare settings, thereby enabling adaptability and innovation. Leaders valued transparency and inclusive decision-making to ensure that diverse perspectives informed the team strategies.

This study indicates that AL is an effective leadership approach for healthcare management in Lithuania. Our findings suggest that healthcare organizations could benefit from AL-based training programs, which may improve leaders’ self-reflective practices, ethical decision-making, and collaboration skills.

## Abstract (it)

Autentiškas vadovavimas (AV) – tai vertybėmis grindžiamas vadovavimo metodas, kurio pagrindiniai akcentai yra savimonė, santykių skaidrumas, subalansuotas informacijos apdorojimas ir suvokta moralinė perspektyva. AV skatina kurti palankią, didelio pasitikėjimo darbo aplinką, kuri stiprina komandos sanglaudą, didina produktyvumą ir etikos standartų laikymąsi. Sveikatos priežiūros darbuotojams tenka dideli iššūkiai ir reikalavimai, susiję su į žmogų orientuota priežiūra, todėl AV tampa vertinga galimybe padidinti vadovavimo ir vadybos veiksmingumą. Šiame tyrime nagrinėjami pagrindiniai AV konstruktų ir galimybių aspektai Lietuvos sveikatos priežiūros sistemos kontekste.

Taikant kokybinį interpretacinį fenomenologinį metodą, buvo atlikti pusiau struktūruoti interviu su vyresniaisiais sveikatos priežiūros vadovais Lietuvoje. Tyrimo tikslas – ištirti, kaip šie vadovai supranta ir praktikuoja AV. Buvo atlikta teminė analizė, remiantis tyrimo dalyvių pusiau struktūruotų interviu išrašais apie AV keturių teorinių dimensijų suvokimą.

Rezultatai parodė, kad Lietuvos sveikatos priežiūros lyderiai savimonę, moralinį sąžiningumą ir prisitaikymą mano esant esminiais veiksmingo lyderiavimo elementais. Lyderiai pabrėžė, kad savirefleksija, atvirumas atsiliepimams ir moralinis nuoseklumas yra esminiai jų vaidmeniui, leidžiantys jiems valdyti sudėtingas etines pareigas ir stiprinti pasitikėjimą organizacijoje. Paaiškėjo, kad AV skatina atsparumą dinamiškoje sveikatos priežiūros aplinkoje, taip sudarant sąlygas prisitaikyti ir inovacijoms. Vadovai vertino skaidrumą ir įtraukųjį sprendimų priėmimą, siekdami užtikrinti, kad komandos strategijos būtų grindžiamos įvairiomis perspektyvomis.

Šis tyrimas rodo, kad AV yra veiksmingas lyderystės metodas, taikytinas sveikatos priežiūros vadyboje Lietuvoje. Mūsų išvados rodo, kad sveikatos priežiūros organizacijos galėtų pasinaudoti AV pagrįstomis mokymo programomis, kurios galėtų pagerinti vadovų savirefleksijos praktikas, etišką sprendimų priėmimą ir bendradarbiavimo įgūdžius.

## Introduction

Substantial evidence indicates that the leaders who foster team environments characterized by positive connections and supportive relationships are more likely to achieve successful outcomes, by inspiring their teams to perform effectively and excel in their work. The literature demonstrates that such environments contribute to team success by strengthening relationships within and between teams [1], increasing levels of trust [2], and promoting higher productivity and a more positive work environment [3]. For individuals seeking to advance their careers and create a lasting impact within their organizations, investing time and effort in developing authentic leadership (AL) practices has been shown to be particularly valuable [3].

AL is a leadership approach that emphasizes self-awareness, relational transparency, internalized moral perspective, and balanced information processing. By aligning actions with personal values, AL supports trust, collaboration, and open communication. Research has demonstrated the positive influence of AL on organizational performance and follower well-being, and a well-developed theoretical framework exists to measure its core components and effects on follower attitudes and behaviors [4,5].

ALs are commonly described as individuals with a positive outlook who engage in honest and open interactions with others. Through such behaviors, they foster trust among their subordinates and generate enthusiasm for shared goals, thereby supporting effective individual and team performance. Previous research has highlighted the value of AL in strengthening workplace relationships and enhancing commitment to an organization’s vision, particularly through its emphasis on ethical standards rather than pure financial outcomes [6].

In the context of ongoing healthcare workforce shortages and increasing challenges related to the recruitment and retention of health and social care professionals, leadership approaches that promote trust, engagement, and ethical practice are particularly relevant. Exploring AL in this setting may offer insights that support leadership development and professional growth among health and social care leaders while also contributing to the broader body of knowledge on the application of AL theory in healthcare contexts [7].

The relevance of AL for leaders in the health, aged, and social care sectors lies in its emphasis on trust, ethical conduct, and employee engagement – i.e., factors that are particularly critical in high-pressure environments centered on patient and consumer well-being. Despite the increasing global interest in AL, little is known about how this leadership approach is perceived and experienced in the Lithuanian healthcare context. Moreover, research focusing on leadership in Lithuania is limited. Existing studies have examined leadership competencies and practices in Lithuanian healthcare organizations [8–10]; however, none have specifically addressed AL or explored how healthcare leaders understand and enact its core principles.

The Lithuanian healthcare system provides a distinctive backdrop for examining AL, shaped by post-Soviet health system reforms, ongoing structural centralization, workforce constraints, increasing administrative demands, and evolving public expectations regarding transparency, accountability, and patient-centered care. In this context, leadership is influenced not only by organizational responsibilities but also by broader cultural and historical factors, including hierarchical management traditions and increasing alignment with European Union governance standards. These contextual elements may shape how leadership authenticity, ethics, and relational transparency are understood and enacted, underscoring the importance of examining AL within this specific sociocultural and health system environment.

**The aim of this study** is to explore how healthcare managers in Lithuania understand AL and how they describe the enactment of its core dimensions in their leadership practice.

**The research question guiding this study is:** *How do healthcare managers in Lithuania perceive and experience AL in their professional roles?*

## Theoretical Background

The development of leadership that prioritizes ethical behavior, employee well-being, and organizational performance has gained increasing attention in the recent literature [11, 12]. AL exemplifies this paradigm, emphasizing leaders who act consistently with their core values and self-concept. Historically, the concept of authenticity can be traced to ancient Greek philosophy, which promoted self-knowledge through the Delphic maxim ‘know thyself’ [13]. AL emerged in the literature in the 1960s as a way to describe how an organization authentically reflects itself through leadership [14]. Luthans and Avolio (2003) note that authenticity has evolved through modernist ideals of self-direction, trustworthiness, and consistency, into postmodern questioning of multiple selves, thereby emphasizing the importance of leaders expressing their ‘real self’ in their actions and decisions [15].

AL is characterized as both a leader’s behavior defining their role within an organization and as a broader organizational phenomenon, meaning that all members in the organization act with authenticity as if they were a single entity through their responses to responsibility ascribed within the organization or their reactions to uncertainty and their innate creativity [16–17]. Authentic leaders are mission-driven, aligning followers with organizational goals while asserting the creation of value beyond financial metrics [11]. Luthans and Avolio (2009) further linked AL to Positive Organizational Behavior, suggesting that authentic leaders encourage positive inquiry and learning, promoting self-development among employees [18].

George and Sims (2007) define five core dimensions of authentic leaders [19], by outlining that authentic leaders:

1. Pursue and display purpose and direction with passion so that people want to follow and show the purpose of leadership.
2. Practice solid values, asserting that, without perceived integrity, trust cannot be established, which negatively affects the followership.
3. Authentic leaders are said to ‘lead with the heart’ and engage the hearts of those they serve, aligning their interests with those of those they lead. An authentic leader requires empathy and compassion for the people they work with, and the courage to make difficult decisions.
4. Authentic leaders establish enduring relationships built on connectedness and a shared purpose of working together towards a common goal.
5. Demonstrating self-discipline is a key behavior of authentic leaders in producing results. Authentic leaders take full responsibility for their outcomes and hold others accountable for their performances.

These dimensions align with the findings of Walumbwa et al. (2008), who validated a multidimensional model of AL comprising self-awareness, relational transparency, internalized moral perspective, and balanced processing across cultures, demonstrating their predictive power for work attitudes and performance [20].

The characteristics of authentic leaders include building legitimacy through honest relationships, valuing their followers’ input, maintaining truthful self-concepts, demonstrating passion, and generating enthusiastic support to improve performance [19,21–23]. Hsiung (2012) extends this understanding by showing that AL influences the employee voice behavior, mediated by positive mood and leader–member exchange and moderated by procedural justice climate, thus highlighting the cross-level impact of AL on organizational outcomes [24].

The characteristics described above indicate that authentic leaders exhibit self-awareness and an ongoing process of reflection on their personal strengths, weaknesses, and values, which ensures that they remain true to their core beliefs. They also practice relational transparency by openly sharing their thoughts and beliefs while minimizing the expression of inappropriate emotions and engaging in balanced processing by actively seeking and considering opposing viewpoints before making decisions. Moreover, authentic leaders maintain an internalized moral perspective, adhere to ethical principles in relationships and decision-making, and remain resilient to external pressures [4,19,21]. A key component of AL is the leader’s self-concept and reflective capacity, which connects these traits to the long-term development. Shamir and Eilam (2005) argue that a leader’s life story – including self-knowledge, self-concept clarity, and person-role merger – forms the foundation of AL development. The life story not only shapes leader behavior but also provides followers with cues to assess the leader’s authenticity, linking personal reflection to perceived credibility [25]

Authentic leaders also integrate positive psychological capacities and organizational contexts to foster self-regulated, positive behaviors. Luthans and Avolio (2003, 2009) highlighted that AL involves confidence, hope, optimism, resilience, transparency, and moral conduct, cascading these behaviors throughout the organization to develop future leaders [15,18]. This approach positions AL at the intersection of positive psychology, transformational leadership, and ethical development, addressing the increasing complexity and turbulence of contemporary organizational environments [15].

Finally, AL interacts with team dynamics. Schaubroeck, Lam, and Xie (2007) demonstrated that leadership behaviors influence the team performance through team potency, moderated by cultural values such as collectivism and power distance, which suggests that AL may enhance collective efficacy by aligning ethical, relational, and motivational processes within teams [26]. This complements the findings of Hsiung (2012) regarding the procedural justice climate and highlights the broader systemic impact of authentic leaders on both individual and group performance [24].

In summary, AL represents a theoretically grounded approach that integrates ethical behavior, self-awareness, relational transparency, positive organizational psychology, and systemic influences on both followers and teams. Its applicability is strengthened by cross-cultural validation, life story approaches, and evidence of employee outcomes such as voice behavior, highlighting its relevance in contemporary organizational research and practice [15,18,20,24–26].

## Materials and Methods

### Study design

A qualitative research design using *Interpretative Phenomenological Analysis* (IPA) was employed to explore how healthcare managers in Lithuania perceive, experience, and make sense of AL in their professional roles [27]. IPA is especially suitable for examining lived experiences and the meanings which individuals assign to them, thereby making it an appropriate methodological approach for exploring leadership perceptions and practices in this study.

### Participants and sampling

Senior leaders and managers from various healthcare organizations in Lithuania were invited to participate in the study to gain insights into AL among healthcare service leaders. Eligible participants were required to hold a managerial position within a healthcare organization and have at least one year of experience in that role. The study included leaders representing different sectors of healthcare, including governmental/state institutions, personal healthcare services, public health services, and non-governmental organizations (NGOs).

Convenience sampling was used to recruit the participants. An initial sample size of six interviews was planned for the preliminary analysis. By the sixth interview, the emergence of new and meaningful insights declined, which indicated that data saturation had been reached [28]. Three additional interviews were conducted to confirm this assessment and ensure thematic stability. Data collection was concluded after nine interviews, as further interviews yielded minimal information.

To ensure anonymity, the participants were assigned identification codes. The sociodemographic characteristics of the participants are presented in Table 1.

**Table 1:** Sociodemographic data of the managers who participated in the interview

Code	Gender	Age	Employment positions
AL_1	Female	36	Director of a municipal hospital
AL_2	Female	58	Director of a public health bureau
AL_3	Male	59	High-level official of the Health Ministry in Lithuania
AL_4	Female	46	Deputy Director of a university-level hospital
AL_5	Male	53	Head of the clinical department at a university-level hospital
AL_6	Male	43	Deputy Director for Hospital Management and Development at a university-level hospital
AL_7	Male	33	General Secretary (CEO) of an NGO
AL_8	Female	44	Director of a public health bureau
AL_9	Female	50	Director of Nursing at a university-level hospital

### Data collection

Data were collected through remote semi-structured interviews. The interview guide consisted of two main sections. The first section focused on the participants’ understanding of AL and their views on the qualities and values that characterize an authentic leader. The second section explored the participants’ personal leadership experiences, values, and leadership practices within their professional roles. Throughout the interviews, the researcher used probing and clarifying questions to encourage reflection and obtain richer and more detailed responses.

The participants were individually contacted to arrange a convenient interview time and were informed that the interviews would be conducted remotely via *Microsoft Teams*. The interview duration ranged from 20 to 59 minutes, with an average length of approximately 30 minutes. All interviews were conducted with the participants’ informed consent and were audio-recorded. Following each interview, the researcher documented observational notes and reflective memos to support the analysis. The audio recordings were transcribed verbatim, and the transcripts served as the primary data source for the analysis.

### Ethical considerations

Written informed consent was obtained from all participants before data collection. Ethical approval for this study was granted by the Bioethics Center of the Lithuanian University of Health Sciences.

### Data analysis

Qualitative content analysis was performed by using a combination of inductive and deductive approaches. Inductive analysis was used to examine the participants’ understanding of AL and the characteristics of authentic leaders. Meaningful words, phrases, and concepts were initially coded and subsequently organized into subcategories based on conceptual similarity. These subcategories were then grouped into broader categories, from which, overarching themes were developed in relation to the research questions.

Deductive analysis was employed to identify leadership behaviors, traits, and experiences corresponding to the four core dimensions of AL: self-awareness, relational transparency, balanced processing, and an internalized moral perspective. Throughout the analytic process, themes were supported by illustrative quotations drawn from the participants’ accounts with the objective to ensure the transparency and credibility of the findings.

## Results

The findings are based on a thematic analysis of nine semi-structured interviews with healthcare managers in Lithuania; they provide insights into how the participants understand and experience AL in their professional roles. The analysis identified themes related to managers’ conceptualizations of AL, as well as how the core dimensions of AL, specifically, self-awareness, balanced processing, relational transparency, and an internalized moral perspective, are reflected in everyday leadership practices. Selected verbatim quotations are presented to illustrate each of the themes.

### Understanding authentic leadership

The participants described AL primarily in terms of personal qualities and behaviors that enable leaders to influence, motivate and engage others. Charisma was frequently emphasized as a key characteristic of AL, particularly in relation to inspiring followership and gaining trust.

“I think it should be a charismatic personality; otherwise, you really won’t be able to sway those people so that they believe in you, that they follow you.” [AL_8]

In addition to charisma, the participants highlighted authenticity as an expression of a leader’s unique personality rather than strict adherence to formal leadership models or textbook definitions.

“Authentic, it is exceptional. It has unique features. It does not reflect all truths written in management and leadership textbooks.” [AL_4]

Authentic leaders were also perceived as innovators who are willing to initiate change, introduce new ideas, and challenge established practices within their organizations.

“He is such an innovative guide, always following the latest news… not afraid to initiate changes.” [AL_1]

### Competencies, qualities, and practices of authentic leaders

The participants identified a range of competencies they considered essential for AL in healthcare settings. Communication skills and the ability to build meaningful relationships were consistently emphasized in the literature.

“Your leadership, your decision-making, your communication, your involvement, and your relationship-building – each action seems to contribute to authenticity.” [AL_7]

Creativity, ethical behavior, and the ability to motivate teams were also highlighted as central to leadership.

“That ideal leader who is creative, team-oriented, ethical, and motivating.” [AL_6]

**Figure 1:**
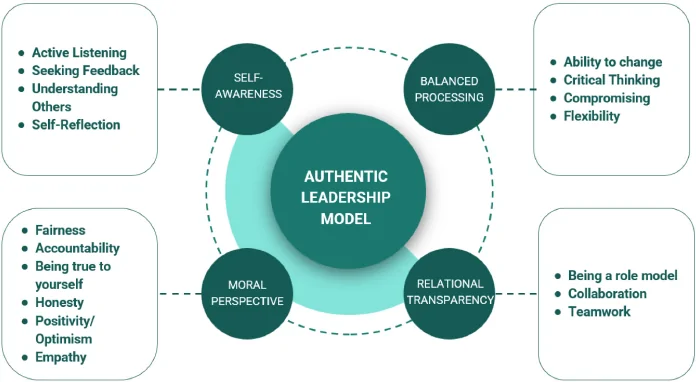
Authentic Leadership Amongst Leaders and Managers in the Health Sector – Study Participants

The participants’ descriptions of their leadership practices were subsequently analyzed in relation to the four core dimensions of AL. Figure 1 presents the thematic structures of these dimensions.

### Self-awareness

Self-awareness emerged as a foundational component of AL. Leaders emphasized the importance of listening to others and understanding different perspectives to achieve effective collaboration within the organization.

“I always participate too, I hear, I listen, and I delve deeper into how people understand things.” [AL_2]

Self-reflection was described as an ongoing process supported by training, self-assessment, and feedback from others.

“I started to recognize my weak points and refined my strong points.” [AL_1]

“…when you start asking not what we do, but why we do it.” [AL_7]

### Balanced processing

Balanced processing was reflected in the participants’ openness to change, critical thinking, and willingness to consider multiple viewpoints. Leaders described the necessity of adapting to evolving organizational and systemic demands of the school.

“What may seem to have been acceptable once, it absolutely does not work nowadays.” [AL_7]

Decision-making was often described as a collaborative process involving dialogue, compromise, and trust in others.

“I compromise more now, and I understand that I am not alone.” [AL_1]

“You need to somehow see that chessboard and anticipate those few moves forward.” [AL_7]

### Relational transparency

Relational transparency was associated with being a role model and demonstrating consistency between values and actions. The participants emphasized leading by example as a key leadership responsibility.

“If you are talking about physical activity, then there must also be an example.” [AL_2]

Collaboration and empowerment of team members were also central to relational transparency. Leaders described supporting others’ development and fostering internal leadership capacities.

“We really want to strengthen our inner leadership so that they feel strong inside.” [AL_8]

### Internalized moral perspective

A strong internalized moral perspective was evident in the participants’ accounts. Honesty, fairness, and consistency were described as essential leadership values.

“I apply the same rules everywhere, regardless of people.” [AL_4]

Leaders also emphasized accountability, authenticity, and the importance of acting in accordance with their personal values to maintain trust.

“If you are dishonest in small things, you will be truly dishonest in big things.” [AL_7]

Optimism and empathy were highlighted as important moral and relational qualities, particularly in the healthcare context.

“Empathy is very important… Employees should not be afraid of approaching the leader.” [AL_9]

### Context-specific leadership characteristics in healthcare

Beyond the core dimensions of AL, the participants highlighted leadership characteristics that they considered particularly important in healthcare settings. The ability to adapt to constant change and make complex decisions was emphasized as critical.

“It is about wanting and being able to change yourself and want to change the team and organization.” [AL_1]

Leaders described motivating their teams through trust, delegation, and empowerment.

“If the manager trusts people, gives them freedom to act… then, the team is motivated.” [AL_8]

Participants also highlighted a strong focus on outcomes, alongside innovation and creativity, as essential for sustaining progress in healthcare organizations.

“That public health change we’re making is endless.” [AL_3]

“It gives the desire to move forward, to bring even more innovations.” [AL_8]

## Discussion

This study examines the qualities and challenges of AL among healthcare managers in Lithuania, offering insights into how authenticity is understood and practiced in healthcare management. A key finding was the importance of self-awareness and self-reflection in leadership development. The participants emphasized that understanding personal and organizational strengths and weaknesses is essential for managing stress, responsibility, and the complexity of healthcare environments.

Ethical behavior, honesty, and fairness were consistently identified as core leadership values, shaped by personal integrity and societal and governmental expectations. Moral integrity was seen as central to shaping the organizational culture and supporting patient-centered care. Participants also highlighted the importance of critical thinking and balanced decision-making, noting that healthcare leaders must often navigate limited resources, competing priorities, and high-risk human services. Effective leadership was described as requiring compromise and thoughtful judgment.

Collaboration and teamwork were viewed as essential for delivering coordinated and safe patient care. Transparency and open communication were emphasized as key mechanisms for building trust within teams and across organizations. These findings were consistently articulated across participants and aligned with previous AL research, suggesting that the core components of AL are well understood and widely shared among healthcare leaders [29,30].

The participants identified a range of traits and skills as particularly relevant to effective AL, including charisma, communication, creativity, empathy, and moral integrity. These attributes reflect a combination of inherent personal qualities and learned leadership skills, consistent with the prior AL literature [30]. The emphasis on these qualities was closely linked to the nature of healthcare as a human service sector, where both direct and indirect patient-centered care are central to the work.

Adaptability and innovation have emerged as particularly important in response to ongoing biomedical, regulatory, economic, and societal changes in the healthcare sector. Leaders, especially those in senior roles, emphasized the need to initiate and manage change while fostering collaboration and innovation within their teams. AL was seen as valuable in supporting resilience, motivation, and engagement in uncertain and dynamic environments, as well as in guiding outcome-oriented and strategic decision-making.

The findings should be interpreted within the specific context of the Lithuanian healthcare system. Following the break-up of the Soviet Union in 1991, Lithuania, like other post-Soviet countries, underwent profound political, economic, and social transformations, including the restructuring of its healthcare system. The inherited Soviet Semashko system, while providing universal coverage, has been criticized for its inefficiency, low responsiveness, and inconsistent quality of care [31]. This created not only the need for structural reforms but also an urgent demand for capable health-system leaders [32]. The challenging nature of the healthcare reform required managers who could adapt, respond to uncertainty, and navigate complex, ambiguous situations. To address this need, the Lithuanian University of Health Sciences (LUHS), in collaboration with international partners under the *TEMPUS* project, developed a postgraduate program specifically aimed at training healthcare managers [33]. It was the first program of its kind not only in Lithuania but also among the former Soviet Union countries. Today, LUHS provides leadership training not only for future public health managers but also for other healthcare professionals, including pharmacists [34].

Within this context, the emphasis placed by the participants in this study on moral integrity, accountability, and adaptability may reflect the ongoing transformation of the Lithuanian healthcare system, where leaders must balance regulatory demands, limited resources, and societal expectations for ethical and transparent governance. Compared to research in other countries, where AL is often linked to empowerment and innovation, the Lithuanian leaders in this study appeared to prioritize fairness, consistency, and personal responsibility. These differences are likely shaped by historical leadership norms, organizational hierarchies, and the evolving nature of the healthcare reform in Lithuania. Therefore, the findings should be interpreted as context-specific insights rather than universally generalizable statements.

Leadership development should occur at all organizational levels to foster collaborative cultures [35]. There is clear evidence that AL is associated with a wide variety of positive outcomes for staff, including job satisfaction, structural empowerment, work engagement, and trust in managers [36]. Consequently, AL has become an increasingly common topic in both undergraduate [37] and postgraduate training programs [38]. This approach could also be applied to healthcare specialist training programs in other countries to strengthen leadership capacity and improve organizational outcomes.

This study has some limitations. The voluntary choice of participants to participate in the study may also have influenced the results of the study, as they may only represent a certain population group with similar values and goals. This could increase the risk of reflecting only a certain opinion without actually revealing other points of view on the investigated issue. Therefore, the results of the study cannot be considered as representing the opinion of the research population (managers engaged in health care activities); the findings only show the attitudes and personal insights of those individuals who participated in the study. Another potential weakness of the study may be the fact that the study used subjective assessment methods and did not make objective observations. Therefore, this study only reflects the opinions and beliefs of the respondents, but these named decisions and actions are not necessarily applied in their real managerial practice.

## Conclusions

This study provides insights into how healthcare managers in Lithuania understand and practice AL. The findings indicate that AL is characterized by self-awareness, relational transparency, internalized moral perspective, and balanced decision-making, which together support trust, motivation, and effective collaboration in complex healthcare environments. A key contribution of this research is its context-specific perspective: Lithuanian leaders emphasized fairness, accountability, and ethical behavior, reflecting historical, organizational, and cultural influences in a transitioning healthcare system. Practically, healthcare organizations can strengthen leadership effectiveness through AL-focused training programs that promote self-reflection, ethical decision-making, and relational skills, supported by mentorship, feedback, and a culture of transparency and collaboration. These findings offer actionable insights for advancing AL research in healthcare and guiding leaders to improve their skills, team relationships, and organizational performance.
